# Allele frequency net database (AFND) 2020 update: gold-standard data classification, open access genotype data and new query tools

**DOI:** 10.1093/nar/gkz1029

**Published:** 2019-11-13

**Authors:** Faviel F Gonzalez-Galarza, Antony McCabe, Eduardo J Melo dos Santos, James Jones, Louise Takeshita, Nestor D Ortega-Rivera, Glenda M Del Cid-Pavon, Kerry Ramsbottom, Gurpreet Ghattaoraya, Ana Alfirevic, Derek Middleton, Andrew R Jones

**Affiliations:** 1 Center for Biomedical Research, Faculty of Medicine, Autonomous University of Coahuila, Torreon, Mexico; 2 Computational Biology Facility, University of Liverpool, Biosciences building, Crown Street, Liverpool, L69 7ZB, UK; 3 Human and Medical Genetics, Institute of Biological Sciences, Federal University of Pará, Brazil; 4 Institute of Integrative Biology, University of Liverpool, Biosciences building, Crown Street, Liverpool, L69 7ZB, UK; 5 Department of Molecular and Clinical Pharmacology, Institute of Translational Medicine, University of Liverpool, Liverpool, UK

## Abstract

The Allele Frequency Net Database (AFND, www.allelefrequencies.net) provides the scientific community with a freely available repository for the storage of frequency data (alleles, genes, haplotypes and genotypes) related to human leukocyte antigens (HLA), killer-cell immunoglobulin-like receptors (KIR), major histocompatibility complex Class I chain related genes (MIC) and a number of cytokine gene polymorphisms in worldwide populations. In the last five years, AFND has become more popular in terms of clinical and scientific usage, with a recent increase in genotyping data as a necessary component of Short Population Report article submissions to another scientific journal. In addition, we have developed a user-friendly desktop application for HLA and KIR genotype/population data submissions. We have also focused on classification of existing and new data into ‘gold–silver–bronze’ criteria, allowing users to filter and query depending on their needs. Moreover, we have also continued to expand other features, for example focussed on HLA associations with adverse drug reactions. At present, AFND contains >1600 populations from >10 million healthy individuals, making AFND a valuable resource for the analysis of some of the most polymorphic regions in the human genome.

## INTRODUCTION

The Allele Frequency Net Database (AFND) was originally designed to provide a freely available resource for the storage of frequency data on the polymorphisms of several immune related genes, including the human leukocyte antigens (HLA) system, killer-cell immunoglobulin-like receptors (KIR), major histocompatibility complex class I chain-related genes (MIC), and a number of cytokine gene polymorphisms (1). These *loci*, which are known to be among the most polymorphic regions in humans, play important roles in the immune system response. Different research areas have investigated these genomic regions due to their importance in donor-recipient matching in solid organ and stem cell transplantation outcome (2,3), response to various infectious diseases or susceptibility to the development of autoimmune diseases (4,5).

In the last decade, there has been a substantial increase in research demonstrating that individuals carrying particular HLA alleles have a greater or lesser risk of developing immune-mediated adverse responses to particular drugs. As such, there has been growing interest in the field of pharmacogenetics research and clinical practice to understand global distributions of HLA alleles for profiling risk (6,7), which can be assisted by healthy populations in AFND. In the last five years, we have also started to capture case and control frequencies (and further statistics) from studies, to help understand HLA-adverse drug reactions (8). HLA population data, sourced from AFND also often underpins anthropology studies (9–11), as well as *in silico* analyses for vaccine development based on epitope prediction (12–14), among many other applications.

There are currently >23 000 alleles that have been reported at the IMGT/HLA database (Release 3.37.0, July 2019) for HLA, and nearly 1000 alleles in the IPD-KIR database (Release 2.8.0, November 2018) for KIR (15,16). The vast majority of frequency records available in AFND (∼90%) corresponds to eight routinely-typed loci *HLA-A, -B, -C, -DRB1, -DQB1, -DPA1* and -*DPB1*, although not all populations have been typed for all these *loci*. Moreover, there has been a recent increase in the number of populations genotyped for KIR and their corresponding HLA-ligands, which are also broadly analysed in disease association studies and transplantation.

The first release of AFND in 2003 consisted of only a few sections and frequencies of HLA alleles/allelic lineages. However, in the last 17 years, many more tools have been incorporated into the database, producing a new release in 2011 and an update in 2015, both published in *Nucleic Acid Research* database issues (17,18). Over the past three years, nearly 100 000 different users from 186 countries have accessed the database. In this article, we describe an update of the available data sets, new developments for data submission and data sharing, as well as introducing a ‘gold–silver–bronze’ (GSB) criteria on data quality.

## DESCRIPTION OF AFND AND SOURCES OF DATA

### Database content

**Figure 1.**
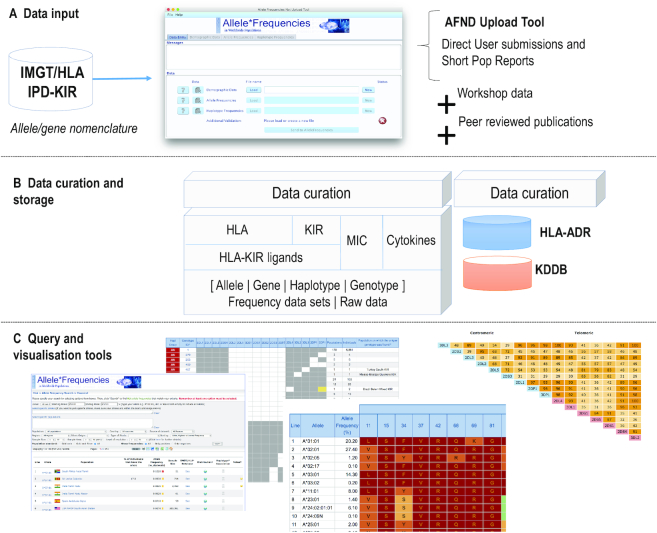
Overview of the main features in AFND. (**A**) Data sets are sourced from short population reports or direct AFND user submissions, both using the upload tool, from International HLA and Immunogenetics Workshops and from other peer reviewed publications, with allele and gene nomenclature synchronized with IMGT/HLA; (**B**) data undergo curation, including classification into gold–silver–bronze quality, and are stored under the different sections of the database; (**C**) a variety of query and custom-built visualization tools allow users to explore the different data sets.

The AFND website collects data from four main sources: (i) data from peer-reviewed publications, (ii) data from populations that are analysed at International HLA and Immunogenetics Workshops (IHWSs), (iii) submissions from individual laboratories across the world, and, more recently, (iv) short publication reports (SPR) in collaboration with the *Human Immunology* journal (19,20), as shown in Figure 1A. As of September 2019, we have compiled information on >1600 healthy populations from >10 million people. The HLA section contains the majority of the submissions with 1228 populations, followed by KIR 256, MIC 64 and 124 populations analysed for cytokine gene polymorphisms (Table 1). Currently, population data sets from 141 countries are included within AFND, with the highest coverage in Europe (396 populations), followed by North America (256 populations), South Asia (249 populations) and South and Central America (232 populations). In this database, users are able to browse for allele, gene, genotype or haplotype frequencies for HLA, KIR, MIC and cytokines, depending on their availability.

**Table 1.** Frequency data sets by polymorphic region at AFND, figures as of September 2019

Polymorphic region	Population studies	Gene/allele data	Haplotype data	Genotype data
HLA	1228	1213	596	157
KIR	256	255	-	171
Cytokine	124	124	-	-
MIC	62	62	23	-
Total	1672	1656	619	171

### Other databases

AFND is currently hosting other resources, beyond data on healthy individuals. The database for HLA adverse drug reactions (HLA-ADR, http://allelefrequencies.net/hla-adr/) comprises nearly 2000 records from more than 100 studies, covering 46 drugs/drug-classes mainly compiled from literature searches. HLA-ADR allows users to query by drug, disease, locus, allele and so on, returning all the recorded data from case-control studies, including *P*-values for significant (and in some cases non-significant) associations. In addition, the KIR and Disease Database (KDDB, http://allelefrequencies.net/diseases/) captures results from disease association studies in KIR with ∼1500 records from more than 270 studies, including auto-immune disorders, infectious disease, cancer and pregnancy-related complications for which studies have demonstrated that the profile of KIR alleles, genes or haplotypes carried by an individual can affect risk. HLA-ADR and KDDB databases have been thoroughly described in two previous publications (8,21). Both HLA-ADR and KDDB can be queried and filtered in various ways, enabling users to assemble data sets for meta-analyses amongst other uses.

### Tools and searching mechanisms

AFND contains a set of different systems to analyse data, as well as searching mechanisms for browsing frequency and genotype raw data. A list of the different tools is shown in Table 2. Despite the number of tools provided in AFND, according to our statistics, healthy population-related searches are among the most popular surveys in AFND with ‘HLA allele frequency search’ covering 27% of the hits in the website, followed by ‘HLA haplotype frequency search’ with 22% from a total of 221k visits over the last three years.

**Table 2.** Overview of the most popular searches and tools available at AFND

Polymorphic region	Tool	Description
HLA	Classical and non-classical allele frequency search	Querying tool to explore allele and phenotype frequencies in one or many populations from the given criteria.
	Haplotype frequency search	Tool to consult a particular haplotype in a set of populations at two or more loci.
	Rare HLA alleles search	Section to ascertain the rarity of HLA alleles based on the confirmation from different databases and individual laboratories.
	Amino acid frequencies in populations	Interactive tool to examine frequencies in a given population comparing alleles differing at amino acid positions.
	Lower resolution search^+^	Tool to search for low resolution data from a given high resolution allele.
	Allele survey^+^	This tool provides a summary of statistical information regarding that allele, based on our Gold Standard populations, which are organized by geographical regions.
	Genotype data browser^+^	Searching mechanism to explore genotype raw data submitted via the short population reports.
KIR	Allele frequency search	Querying tool to explore allele and phenotype frequencies in one or many populations from the given criteria.
	Genotype frequency search	Tool which allows users to display genotype frequencies of a given profile.
	Cell-lines and CEPH families	Section that provides a reference of KIR cell-lines and Centre d' Etude du Polymorphisme Humain (CEPH) families typed at allele level.
	Linkage disequilibrium (LD) analysis^+^	Tool for the analysis of LD among KIR genes by population, country, geographical region and ethnicity.
Cytokines	Cytokine frequency search	Querying tool to explore allele frequencies in one or many populations from the given criteria.
MIC	Allele frequency search	Querying tool to explore allele and phenotype frequencies in one or many populations from the given criteria.
	MIC-HLA association frequency search	Section that provides frequencies of MIC and HLA associations.
Online submissions	Populations	Online submission tool for a new population.
	Confirmation of rare alleles	Section for the confirmation of rare HLA alleles.

^+^New tools and searches since 2015.

### Data quality, submissions and data sharing

Although all submissions by contributors are considered for inclusion, AFND introduces minimal criteria for HLA and KIR populations before they become publicly accessible on the website (Figure 1B). These minimum requirements include validation of allele names, which comply with the IMGT/HLA and IPD-KIR official nomenclature guidelines, homogenization on the naming of the populations, an appropriate assignment of the geographical region to which the population belongs and validation of frequency data using the AFND Upload Tool for SPR submissions. A full list of requirements can be consulted at http://allelefrequencies.net/quality.asp.

Since 2015, AFND has also started receiving submissions for SPR in which the majority of the data corresponds to two-field (or greater) according to the IMGT/HLA nomenclature (Table 3). For this new section users are able to download the submitted data in CSV files. In addition, KIR genotype raw data can be downloaded as CSV files.

**Table 3.** Genotype submissions data to AFND by geographical region and level of resolution

Geographical region	Countries	Individuals	≥ Two-field resolution (%)	Different alleles^a^
South and Central America	8	34 334	46	362
Europe	11	32 856	96	458
South-East Asia	4	14 790	100	187
North-East Asia	1	3568	97	70
South Asia	2	3392	100	212
North America	1	496	100	75
Sub-Saharan Africa	2	359	100	114
Oceania	1	199	100	26
Western Asia	1	52	100	43
Total	31	90 046		

^a^Number of different alleles at two-field resolution.

### Gold, silver and bronze (GSB) standard HLA data sets

**Figure 2.**
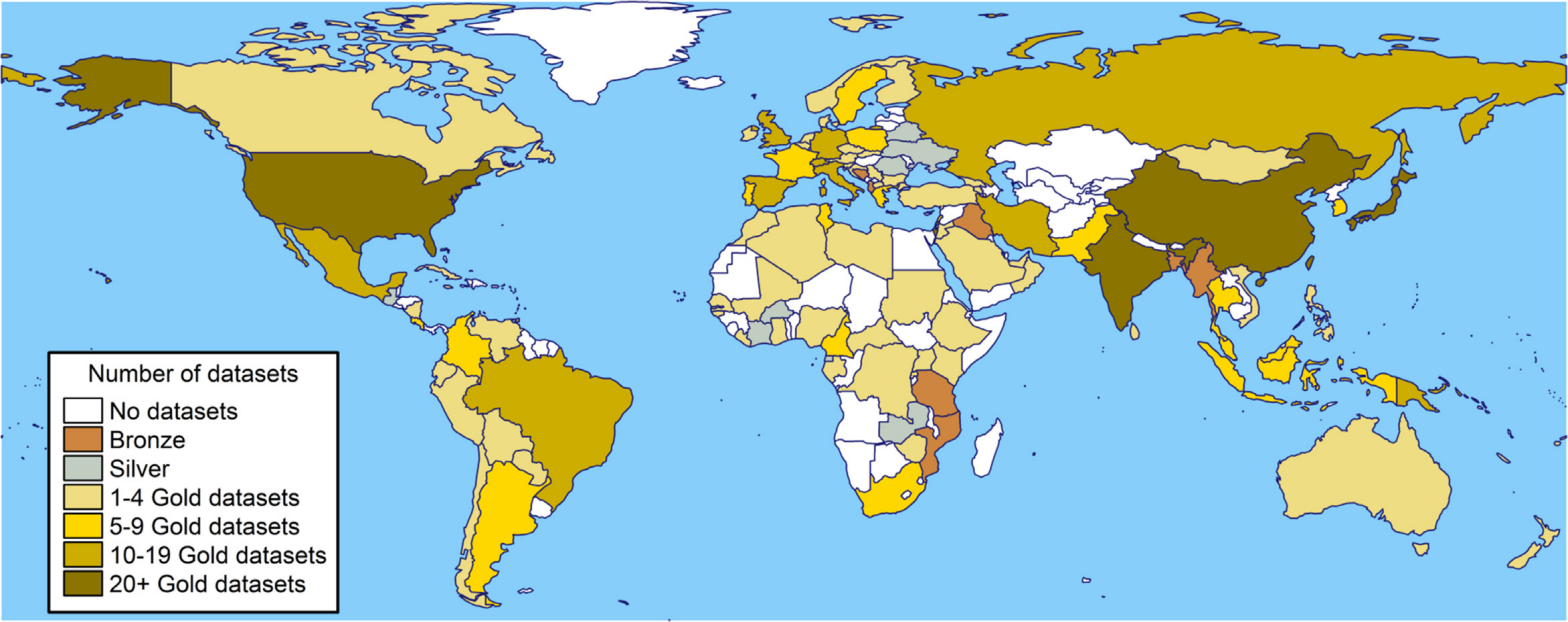
Map showing the distribution of countries with data available for at least one classical HLA locus. Countries with no frequency/genotype data are shown in white; countries with only silver and/or bronze standard populations are shown in grey; gold standard populations are shown in light yellow (1–4 populations with gold standards), yellow (6–9), dark yellow (10–19), brown (≥20).

In order to allow users to browse data sets according to their needs, HLA populations were classified into three groups based on the sample size, level of resolution and frequencies: gold, silver and bronze standards. Three important criteria (allele frequency sums to 1 [±1.5%], sample size >50, two-field resolution) define the ‘Gold Standard’ data set. A ‘Silver Standard’ data set is determined via less stringent criteria with regards sample size (i.e. any sample size) and any level of resolution (but population frequency still sums to 1), for example included to allow users to consult data from a particular geographical region or country if this is not covered by the gold standard data. Finally, ‘Bronze Standard’ includes populations that do not meet these criteria, but remain in AFND as a record of published data sets which are likely to require some care when interpreting. The Bronze Standard set includes all data at one-field resolution for which very large sets exist, for example from Bone Marrow Donor Registries. Following these criteria, AFND has organized all datasets into these categories, resulting in coverage of many countries by several Gold Standard populations, as shown in Figure 2. Figure 2 does however demonstrate that some regions of Africa and Central Asia still do not have any data coverage, and thus we would especially encourage submissions from these countries.

## FUTURE DEVELOPMENTS

We are currently expanding the offering in AFND to add tools to assist in clinical transplantation settings. In the UK, 393 (8%) of adult patients on the kidney transplant waiting list have waited over 5 years for an offer and over 80% are classified as ‘highly sensitized patients’ with HLA-specific antibodies directed towards at least 85% of donors. Such patients also suffer increased morbidity and mortality compared to less sensitized patients. Antibodies are often assigned using single antigen bead (SAB) assays but there remains much debate as to the clinical significance of antibodies detected solely by this method and the clinical significance of the quantitative threshold used to determine the presence of a given antibody. We will shortly be releasing the AbDelister tool, which allows users to filter a patient's longitudinal SAB-defined HLA-specific antibodies, altering the quantitative threshold used and time intervals into levels. Up to five filter levels can be set for each locus and antibodies within each can be removed from a patient's profile to test the potential for increasing the percentage of donors from which the patient could receive a kidney. We welcome feedback and testing of the AbDelister tool.

AFND will continue to develop over the coming years via the addition of more data, and providing new querying mechanisms, for example in response to user requests. We also wish to provide capabilities for performing statistical analyses online as well as incorporation of other applications for data visualization, for example allowing populations to be clustered. In addition, we are currently including support for nomenclature that describes ambiguity in the typing (such as ‘G’ and ‘P’ codes) for HLA alleles, via the AFND Upload tool. Finally, we are also moving into a new website version which will include a set of tutorials and documentation of the data sets and tools available.

## Notes

Present address: Institute of Integrative Biology, University of Liverpool, Biosciences building, Crown Street, Liverpool L69 7ZB, UK.
